# The Many Faces of Astrocytes in Alzheimer's Disease

**DOI:** 10.3389/fneur.2021.619626

**Published:** 2021-08-31

**Authors:** Michael D. Monterey, Haichao Wei, Xizi Wu, Jia Qian Wu

**Affiliations:** ^1^The Vivian L. Smith Department of Neurosurgery, McGovern Medical School, The University of Texas Health Science Center at Houston, Houston, TX, United States; ^2^Center for Stem Cell and Regenerative Medicine, UT Brown Foundation Institute of Molecular Medicine, Houston, TX, United States; ^3^MD Anderson Cancer Center UTHealth Graduate School of Biomedical Sciences, Houston, TX, United States

**Keywords:** neurodegeneration, Alzheimer's disease, biomarkers, reactive astrocyte, heterogeneity, single-cell sequencing, neuroinflammation

## Abstract

Alzheimer's disease (AD) is a progressive neurodegenerative disease and is the most common cause of dementia in an aging population. The majority of research effort has focused on the role of neurons in neurodegeneration and current therapies have limited ability to slow disease progression. Recently more attention has been given to the role of astrocytes in the process of neurodegeneration. Specifically, reactive astrocytes have both advantageous and adverse effects during neurodegeneration. The ability to isolate and depict astrocyte phenotype has been challenging. However, with the recent development of single-cell sequencing technologies researchers are provided with the resource to delineate specific biomarkers associated with reactive astrocytes in AD. In this review, we will focus on the role of astrocytes in normal conditions and the pathological development of AD. We will further review recent developments in the understanding of astrocyte heterogeneity and associated biomarkers. A better understanding of astrocyte contributions and phenotypic changes in AD can ultimately lead to more effective therapeutic targets.

## Introduction

Alzheimer's disease (AD) is the most common form of dementia worldwide and was first described over 100 years ago by Alois Alzheimer. Alzheimer's disease is a prominent disease throughout the world with a significant impact on the health care system, estimated at nearly $500 billion annually (1). Currently, the FDA has approved few drugs for AD, which aim to improve quality of life but do not change or slow disease progression (2).

At this time the pathophysiological mechanisms of AD are not fully understood, and current therapeutic interventions are limited in efficacy. The pathological hallmark of the disease is the deposition of beta-amyloid (β-amyloid) plaques and the resulting formation of neurofibrillary tangles composed of hyperphosphorylated tau protein (3). Due to the location of these pathological markers within neurons, neurons have been the target of research. Ramon y Cajal eloquently demonstrated these pathological hallmarks decades earlier (4). Interestingly, Ramon y Cajal also noted reactive hypertrophic astrocytes that surrounded senile plaques and blood vessels with amyloid deposits in post-mortem AD patients (4). Thus, astrocytic changes due to neurodegeneration are not a new discovery. However, there has been minimal advancement in understanding the role of astrocytes in the development of AD. This lack of progress was likely due to insufficient technology and methods. Due to new innovative technologies, there is an increasing focus on elucidating the physiological changes within astrocytes during AD progression.

The astrocyte is a prevalent cell type within the central nervous system (CNS). They have diverse and vital functions within the CNS including contributions to synaptogenesis, ion homeostasis, neurotransmitter buffering, the blood brain barrier (BBB), and inter/intracellular communication (5). Furthermore, astrocytes are a heterogeneous group of cells with diverse phenotypes and functions specific to their origin regionally (5, 6). Currently, significant effort has been dedicated to investigating the distinct functions of astrocytes as it relates to neurodegenerative disease (5, 7).

This review will examine the current understanding of the roles of reactive astrocytes and potential astrocytic biomarkers unique to AD. We will further explore new technologies such as single-cell sequencing and its potential effectiveness in deciphering the phenotypic changes astrocytes undergo in the context of AD. Finally, we will examine how these technologies can help to dissect astrocyte states or subtypes during AD progression.

## Alzheimer's Disease Pathology

Alzheimer's disease is an irreversible brain disorder that slowly destroys memory and thinking skills. Two forms of AD exist, familial, and sporadic. Familial AD accounts for <5% of cases and is associated with three subtypes defined by unique genetic mutations (8). The first unique genetic mutation involves the amyloid-beta precursor protein (APP) gene, which controls the formation of the amyloid precursor protein. The APP role is not fully understood, but it is suspected that it helps direct the migration of neurons during early development (9). Mutations cluster around the γ-secretase cleavage site of APP, resulting in longer and more fibrillogenic β-amyloid (10). Two other genes implicated in familial AD are presenilin1 (*PSEN1*), and presenelin2 (*PSEN2*) (11). These genes encode for subunits of a complex of gamma (γ)-secretase, which is involved in the proteolysis and processing of APP. The sporadic or late-onset (>65 years old) of AD lacks a complete explanation for its development. However, there is a host of risk factors associated with the onset of the disease. For example, there is a genetic association of carriers of the Apolipoprotein E4 (*APOE4)* allele, Clusterin, and mutations in triggering receptor expressed on myeloid cells 2 (*TREM2*) (12, 13). Other risk factors for the development of sporadic AD are associated with both environmental and modifiable lifestyle factors (14, 15).

The gold standard of pathologic diagnosis of AD includes extracellular amyloid plaques and intracellular neurofibrillary tangles. Amyloid plaques aggregate within the isocortex and are found in all six cortical layers (16). β-amyloid deposition and plaque formation are accompanied by reactive astrogliosis and microglial activation (17). It has been shown in post-mortem specimens that neurofibrillary tangles were densely associated with those areas of the brain most affected by the disease, such as the hippocampus (18). The number of these tangles is correlated with severity of symptoms (19). Tau protein is a microtubule-associated protein (MAP) which aggregates into neurofibrillary tangles. It is necessary for the function and development of the nervous system and regulation of the normal function of neurons (20). In AD, tau aggregation secondary to post translational changes such as hyperphosphorylation, truncation, glycation, glycosylation, nitration, and ubiquitination results in the formation of neurofibrillary tangles in neuronal cytoplasm (20). For example, in AD, hyperphosphorylation of tau protein is produced by glycogen-synthase-kinase 3β, cyclin-dependent kinase 5 (CDK5), mitogen-activated protein kinase (MAPKs), Fyn, and many others (20). In addition, decreased phosphatases (which dephosphorylate tau) have been found in AD post-mortem specimens. A major phosphatase implicated in AD is protein phosphatase 2 (PP2A). Protein phosphatase 2 inhibition has been shown to increase tau hyperphosphorylation and has been demonstrated to be reduced in AD human brain specimens (20, 21). Thus, the imbalance of kinases and phosphatases together results in hyperphosphorylated tau and progression in AD.

Currently, the approved treatment for AD is directed at controlling symptoms. Further investigation is underway to evaluate possible disease modifying agents to attempt to slow the progression of the disease. Continued research efforts are required to clarify the pathological progression of AD and thus provide new targets for therapeutic development.

## Role of Normal Astrocytes

**Figure 1:**
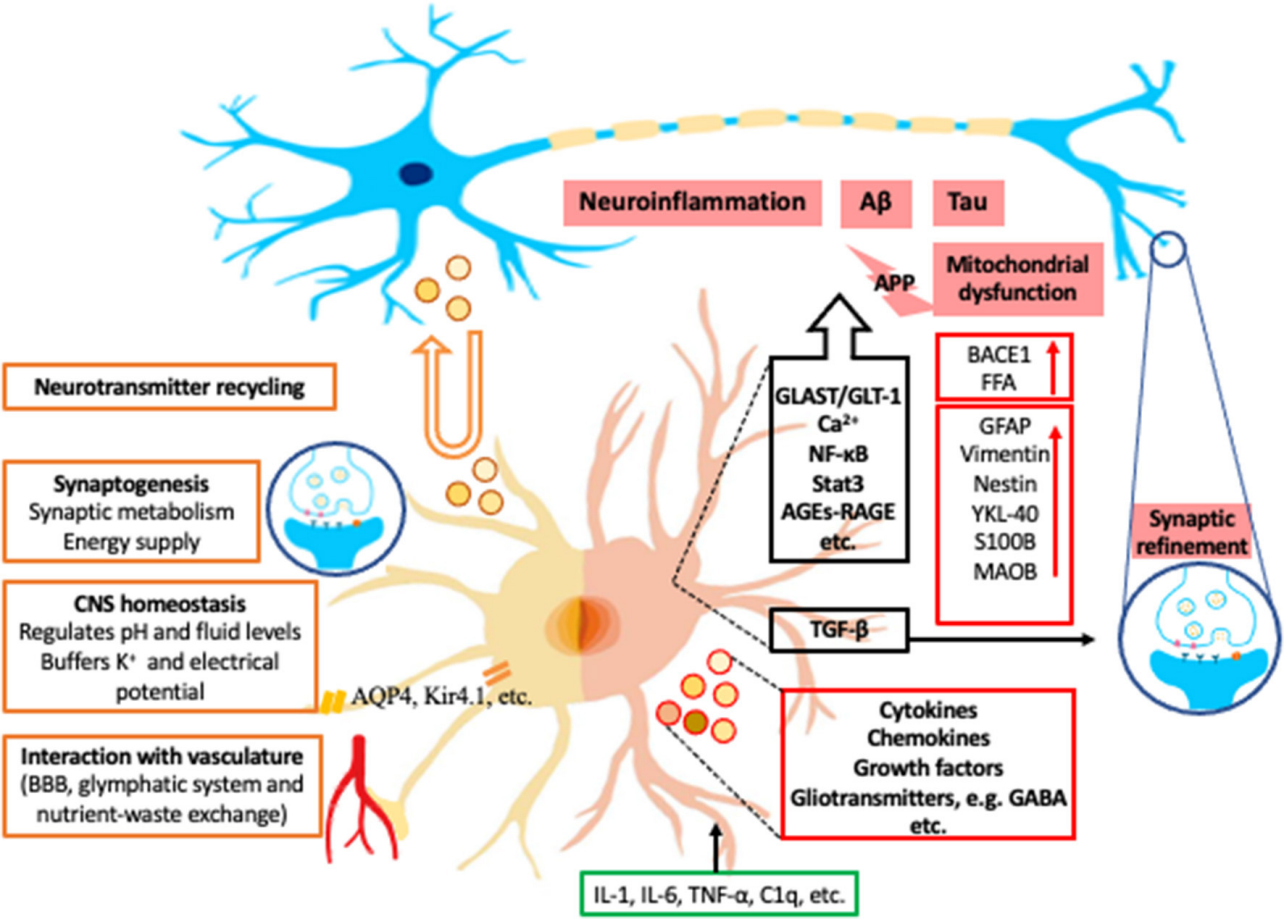
Examples of roles of astrocytes in normal condition and in AD. The left half of astrocyte (yellow) depicts normal astrocyte functions, including neurotransmitter recycling (U-turn arrow and brown-outlined circles), synaptogenesis, central nerve system (CNS) homeostasis, and interaction with vasculature. The right half of astrocyte (Pink) depicts a reactive astrocyte in Alzheimer's Disease (AD). The black box contains some examples of the astrocytic signaling pathways related to AD. Green box indicates examples of inducing factors of reactive astrocytes from microglia. Red boxes are the representative intracellular and secretory molecules (red-outlined circles) and astrocytic makers expressed by reactive astrocytes in AD. Blue circles with icons are synapses. K^+^, potassium; AQP4, aquaporin-4; Kir4.1, inwardly rectifying K^+^ channel subunit 4.1; APP, amyloid-beta precursor protein; BACE1, β-site APP-cleaving enzyme 1; FFA, free fatty acid; GFAP, glial fibrillary acidic protein; YKL-40, chitinase-3 protein like-1; S100B, S100 calcium-binding protein B; MAOB, monoamine-oxidase-b; GLAST, glutamate aspartate transporter; GLT-1, glutamate transporter 1; Ca^2+^, calcium; NF-κB, nuclear factor- kappa B; Stat3, signal transducer and activator of transcription 3; AGEs, advance glycation end-products; RAGE, Receptor for advanced glycation end products; TGF-β, transforming growth factor beta; GABA, gamma-aminobutyric acid; IL, interleukin; TNF-α, tumor necrosis factor alpha; C1q, complement component 1q.

Astrocytes are specialized glial cells and have important roles within the CNS. They are essential to allow the brain to function as an organ and computational structure. Astrocytes have long been postulated and expanded upon since they were histologically depicted by Ramon y Cajal and his contemporaries (22). Initially it was believed that the astrocytes' role within the CNS was structural support for neurons. However, over 100 years ago, Ramon y Cajal found morphological heterogeneity of astrocytes (22). He described nine different morphological subtypes of astrocytes, which led to the development of multiple theories of the vital function of astrocytes. Unfortunately, due to the lack of technology these theories were left largely unproven and forgotten over the next century. More recently new methodologies have revealed that astrocytes execute a variety of essential functions including contributions to the BBB, synaptogenesis, ion homeostasis, neurotransmitter buffering, and the secretion of neuroactive agents (5) (Figure 1).

The understanding of the functional roles in cellular physiology first begins with understanding unique morphological characteristics. Typical protoplasmic astrocytes demonstrate a characteristic spongiform morphology (22). These astrocytes are ubiquitous throughout the gray matter. The astrocyte soma has numerous major branches with multiple secondary and tertiary branches that ultimately form interactions with other neurons and several synapses (23). Astrocytes regulate these synapses by secreting neurotransmitters to target pre and post synaptic sites and modulating function of adjacent neurons and astrocytes (24). This led to the development of the tripartite synapse, which is composed of an astrocyte and two neurons as a functional unit (24). The numbers of synapses an astrocyte interacts with are variable between circuits, brain regions and species. For example, a single astrocyte in the dorsolateral striatum can interact with 50,000 synapses while in the hippocampus stratum radiatum interactions can exceed 100,000 (5). This suggests that the morphological diversity of astrocytes is also related to their location within the CNS (6). The mechanism controlling the morphology of astrocytes remains ill-defined. More recently studies have proven that the loss of connexins and neuroligins alters astrocyte morphology, influencing synapse formation (25, 26). For example, Stogsdill et al. found that the morphological complexity of astrocytes relies on direct contact with neurons mediated by astrocyte neuroligin/neurexin interactions (26). Moreover, changes in astrocyte morphology in response to a pathological insult are ubiquitously noted in variety CNS diseases (27).

As stated, astrocytes are a mainstay in the development of the synapse. At a synaptic level, astrocytes have a plethora of roles to maintain normal synaptic activity. Astrocytes have known roles in synaptic metabolism and are implicated in glycogen processing and storage (Figure 1). Astrocytes can synthesize glycogen, provide glycolytic components for neurons during periods of high demand, and remove free radicals (28). Additionally, when neurons have further energy requirements, glycogen stores can be metabolized to form lactate which is transported from astrocytes to neurons via various monocarboxylate transporters (MCTs) (29).

A new waste clearance system formed by normal astrocytes has been discovered, deemed the glymphatic system (30). In this system, perivascular channels promote the efficient elimination of soluble proteins and metabolites, including β-amyloid, from the CNS. Astrocytes directly contact the CNS vasculature via their end feet and vasodilate or constrict to accommodate nutrient-waste exchange for neurons based on activity (5). For example, astrocytes highly express aquaporin-4 (AQP4) at the end foot processes and in turn can regulate extracellular matrix as well as cell membrane potential (Figure 1) (31). Aquaporin-4 is also vital in the clearance and exchange of solutes in a water dependent manner between the cerebral spinal fluid (CSF) and interstitial fluid (31). Glymphatic dysfunction has been demonstrated in animal models of traumatic brain injury, AD, and ischemic disease, most likely related to dysfunction of AQP4 (32).

Moreover, CNS homeostasis is maintained by astrocytes by regulating pH and fluid levels in the brain, buffering potassium, and recycling neurotransmitters (Figure 1). For example, astrocytes possess a Kir4.1 potassium channel (33). In conjunction this allows astrocytes to control action potential firing (Figure 1) (34).

Overall, the roles of astrocytes are diverse and fundamentally important in the CNS. Therefore, any disruption regarding the normal roles of astrocytes can result in morphological and functional changes that result in pathological consequences. We will further review astrocyte function and the impact on AD.

## Astrocytes in Alzheimer's Disease

Within the CNS astrocytes have a vital role in protecting and repairing neuronal damage (35, 36). Astrocytes respond to inflammatory substances and undergo a process known as reactive astrogliosis (34, 37). Astrocytes become reactive in response to multiple pathological conditions including acute injuries and progressive disorders such as tumor and AD (37). For example, Das et al. completed a meta-analysis of published astrocyte transcriptomic datasets in both acute and chronic neurodegenerative models, which displayed differing genetic astrocyte signatures (38). Specifically, in acute models, astrocytes were found to have upregulated expression for genes involved in protein synthesis, protein degradation, and antioxidant defense, whereas downregulated expression were noted for genes regulating chromatin structure and transcriptional repressors. Conversely, in chronic models, astrocytes were found to have upregulated expression for genes associated with extracellular matrix proteins and downregulated expression for genes associated with glycogenolysis, immune modulation, and antioxidant defense. Thus, astrocytes have unique genetic signatures in response to acute and chronic neurodegeneration.

Inflammation plays a prominent role in the development of AD (17). Reactive astrocytes release molecules including cytokines, chemokines, growth factors, and gliotransmitters (39). Astrocytes also release axon growth-promoting factors essential for axon growth and synaptic formation and maturation in response to injury (34, 40). Additionally, astrocytes increase neuronal viability and mitochondrial biogenesis, protecting neural cells from oxidative stress and inflammation induced by amyloid peptides (41).

The central hypothesis regarding the etiology of AD is that β-amyloid and neurofibrillary tangles produce acute inflammation which activates microglia, the primary inflammatory cells of the CNS, to release inflammatory mediators. This chronic inflammation results in neuronal dystrophy and ultimately leads to the clinical symptoms of AD. More recently, the role of astrocytes in the neuroinflammatory process has been closely evaluated (42, 43). For example, Orre et al., identified differentially expressed genes (DEGs) (807 upregulated and 571 downregulated genes) in AD astrocytes in APPswe/PS1dE9 double transgenic mouse model compared with WT mice (42). These up-regulated genes were enriched in inflammatory response, such as “defense response” and “positive regulation of immune response” and down-regulated genes were enriched in the regulation of synaptic transmission, neurogenesis, and brain and neuron development. Studies demonstrate that reactive astrocytes are induced by activated microglia that release IL-1alpha (IL-1α), IL-1beta (IL-1β), IL-6, tumor necrosis factor-α (TNF-α), and complement component 1q (C1q) (44–46). Furthermore, these cytokines can activate β-secretase and γ-secretase activity, cleaving APP, and stimulating β-amyloid formation by astrocytes, thereby supplementing neuronal β-amyloid production (47). For instance, Zhao et al. (48) demonstrated that primary astrocytes taken from mice and treated with a combination of INF-γ and TNF-α or IL-1β induced the secretion of β-amyloid. β-site APP-cleaving enzyme (BACE1) is an enzyme that works in conjunction with γ-secretase in cleaving APP to form β-amyloid. Previously it was thought that only neurons expressed BACE1, thus being the only cell capable of producing β-amyloid (35). Recent studies on post-mortem AD human brains have exhibited that astrocytes express BACE1 levels high enough to secrete β-amyloid (49). The mouse models that overexpress APP with the Swedish mutation (which is a mutation adjacent to the beta-secretase cutting site in the APP gene), displayed increased expression of BACE1 in correlation with elevated β-amyloid in reactive astrocytes, but BACE1 was not detectable by staining in resting astrocytes in the same mouse model (50). Therefore, inflammatory stimulation of astrocytes can induce BACE1 and in turn secrete more β-amyloid resulting in the progression of AD.

Another important protein associated with astrocytes and AD severity is S100B. During fetal development, it functions as a neurotrophic agent (51). S100B has been shown to induce astrocytes to become reactive in transgenic mice that overexpress *S100b* (52). Further studies have proven that cells, particularly astrocytes, that are S100B positive were located in higher concentrations around neuritic plaques in post-mortem AD brains (19). Specifically, there was a high concentration of these cells in areas of the brain known to be affected severely by AD, such as the hippocampus. The antiprotozoal medication pentamidine, which directly blocks S100B activity, has been studied in an AD mouse model (53). Pentamidine reduced GFAP, S100B and the receptor for advanced glycation end products (RAGE) protein expression, which are implicated in the neuroinflammatory response of astrocytes (53). Cirillo et al. also displayed the neuroprotective effect of pentamidine in CA1 pyramidal neurons (53). Thus, S100B is an important inflammatory regulator of astrocytes involved in phenotypic changes and progression of AD pathology.

Astrocytes are the primary source of cholesterol and lipid production and metabolism, and aberrant cholesterol processing has been implicated in AD development (54, 55). ATP-binding-cassette transporter 1 (ABCA1) is expressed on astrocytes and important in the lipidation of APOE. When cholesterol is abundant, neurons produce β-amyloid to suppress the expression of the ABCA1, which results in increased deposition of β-amyloid (55). Increased free fatty acids have been suggested as a risk factor in the development of AD and high fat diets in animal models resulted in the accumulation of β-amyloid and plaque formation (54, 55). Ceramide, a metabolite of fatty acids, is increased in AD post-mortem brains and β-amyloid production (55). Furthermore, elevated ceramide levels have been shown to induce astrocytes to produce inflammatory cytokines; in turn this activated BACE1 activity and thus β-amyloid production in neurons (55). Therefore, cholesterol and fatty acids prompts astrocyte inflammatory response and further progression of AD.

Additionally, astrocytes recycle glutamate and GABA into glutamine, via glutamine synthetase (56). The glutamine from astrocytes is then used by neurons to produce more glutamate and glutathione (57), thus, providing additional nutrients and protection against reactive oxygen species. Glutamate Transporter (GLT-1) and Glutamate Aspartate Transporter (GLAST) are responsible for 90% of astrocytic glutamate uptake in the brain and are ubiquitous marker for astrocytes (57). In post-mortem AD brains and animal models mRNA of both *Glt-1* and *Glast* are reduced (Figure 1) (58). Astrocyte glutamatergic dysfunction, specifically GLT-1, is associated with the microenvironment of β-amyloid plaques in animal models. For example, when a mouse model lacking one allele of *Glt-1* is crossed with mice expressing mutations in APP and PS1, it accelerated memory impairment and increased β-amyloid (59). Additionally, β-amyloid oligomers and preplaque β-amyloid species have been demonstrated to decrease GLT-1 and GLAST in cultured astrocytes (60, 61). Thus, aberrant glutamate transport results in the disruption in the clearance of excitatory neurotransmitters and increased levels of β-amyloid and tau from astrocytes (62).

Aberrant gliotransmitter released by reactive astrocytes has been suggested as a possible role in AD symptomology, specifically memory loss (63). GABA is a major inhibitory neurotransmitter within the CNS. GABA is metabolized within astrocytes by GABA transaminase to succinate, entering the Krebs cycle, and used for energy production (64). Jo et al. displayed *in vivo* that reactive astrocytes produce GABA via MAOB and release GABA through the bestrophin-1 channel (63). GABA and MAOB content has been noted to be elevated in AD patients and mouse models (63, 64). The excessive GABA produced and released by reactive astrocytes results in activation of neuronal GABA receptors, which results in inhibition of glutamate release, and suppresses astrocytes' pro-inflammatory response (64). However, other studies have demonstrated decreased GABA levels in multiple areas of the brain in post-mortem AD samples (65). Although there are inconsistencies in how GABA influences AD progression, it is clear that GABA dysfunction within astrocytes is involved in AD pathogenesis.

Cellular senescence has been considered as a primary inducing factor of age-associated neurodegenerative disorders, and astrocytes can undergo stress-induced premature senescence (66). Recently, astrocytes have been shown to have decreased normal physiological function and increased secretion of senescence-associated secretory phenotype (SASP) factors in AD, which contribute to β-amyloid accumulation, tau hyperphosphorylation, and neurofibrillary tangle deposition (66). Senescent astrocytes share many similar phenotypes to reactive astrocytes, and it has been suggested that prior studies that focused on reactive astrocytes may have been focusing on senescent astrocytes (66). However, the topic of cellular senescence and its involvement in the development of neurodegenerative disease is controversial at this time (37). In order to verify if senescent astrocytes become reactive in the development of neurodegenerative disease, a significant amount of investigation remains. Specifically, defining molecular markers of normal aging astrocytes over multiple brain regions and compare with reactive astrocytes in neurodegenerative disease will be required (37).

## Signaling Cascades Associated With Astrocytes in AD

There are many molecules and signaling pathways that have been implicated in astrocytes in AD. We review some examples as the following. Astrocyte calcium regulation is regulated by a diverse set of stimuli that can alter intracellular levels. The pathological accumulation of β-amyloid results in inflammatory facilitators, such as bradykinin, to increase intracellular calcium via nicotinic receptors and the P13K-Akt pathway in cultured astrocytes (67, 68). Additionally, β-amyloid has the unique ability to interact with multiple astrocyte cell surface receptors, such as P2Y1, nicotinic receptors, and glutamate metabotropic mGlut receptor, increasing intracellular calcium (Figure 1) (69). Furthermore, Chiarini et al. (70) showed β-amyloid can bind to the calcium sensing receptor (CaSR) in human astrocytes, activating intracellular signaling, which resulted in the production and release of phosphorylated tau. Overall, there is sound evidence that calcium dysregulation is involved in the progression of AD. However, the receptors involved need further investigation to determine their diverse function and ability to be developed as a therapeutic target.

Another signaling cascade important in astrogliosis in AD is nuclear factor-kappa B (NF-κB). Nuclear factor-kappa B is a common transcription factor present in almost all cell types and has a critical function in numerous cellular processes. In the CNS, NF-κB requires strict control to ensure normal neuronal development and function (71). Abnormal NF-κB activation has been previously reported in multiple neurodegenerative diseases, including AD (72). Studies in rat models and post-mortem AD brains have shown an association of NF-κB with β-amyloid (73, 74). Specifically, NF-κB has been shown to have increased activity in neurons, astrocytes, and microglia due to exposure to β-amyloid. This activation results in induction of target genes in reactive astrocytes which induces astrocytes' morphological and functional changes. Nuclear factor-kappa B activation in reactive astrocytes is associated with elevated mitochondrial oxidative metabolism, limiting the supply of pyruvate substrate for neurons (75). The increased production of inflammatory substrates also influences neurons by inducing neuronal oxidative stress and apoptosis (72). The inhibition of NF-κB activation in AD mouse models has been demonstrated to slow the AD pathology and improve neuronal survival and cognition, implicating that the use of NF-κB antagonists could provide therapeutic benefit (76, 77).

Signal transducer and activator of transcription 3 (STAT3) is a transcription factor that is activated through phosphorylation by Janus Kinases (JAK) in response to cytokines, growth factors, and intracellular mediators and has been implicated in the activation of astrocytes (78, 79). Ben Haim et al. showed that STAT3 is activated in reactive astrocytes of several murine and primate AD and Huntington's disease models (79). Conversely, two studies have been completed in AD mouse models with *Stat3* inactivation in astrocytes (78, 80). Ceyzeriat et al. demonstrated that inhibition of STAT3 *in vivo* resulted in reducing amyloid deposition, restoring synaptic deficits, and improved spatial learning (80). Similarly, Reichenbach et al. showed *Stat3* inactivation in astrocytes reduced plaque deposition and improved memory. However, it was also demonstrated that there was a reduction in pro-inflammatory cytokine activation (78). Altogether, these studies provide strong evidence of the potential for targeting STAT3 in astrocytes to slow the progression of AD (37). However, further investigation is required to determine the time point in which STAT3 activation in astrocytes results in pathological consequences.

Receptor for advanced glycation end products is a multi-ligand receptor of the immunoglobulin superfamily of cell surface molecules. They bind advance glycation end-products (AGEs) which are non-functioning glycated proteins or lipids that become glycated after exposure to sugars (81). Advance glycation end-products are associated with aging and have been implicated in neurodegenerative diseases such as AD (82, 83). Additional research has shown that AGEs form early in disease process of AD (84). Engagement of AGEs-RAGE converts a brief pulse of cellular activation to sustained cellular dysfunction and tissue destruction (85). Increasing expression of RAGE on the membranes of neurons and microglia is relevant to the pathogenesis of neuronal dysfunction and death of AD (86). Most pertinent to this discussion is the role of RAGE regarding astrocytes response. Reactive astrocytes surround the β-amyloid plaques and express RAGE (19). It has also been reported that β-amyloid can bind and activate RAGE on astrocytes and induce a pro-inflammatory state via a NF-κB pathway (87). Thus, targeting RAGE has the potential to reduce downstream inflammatory effects.

Transforming growth factor beta (TGF-β) is expressed ubiquitously within the CNS. During development, Transforming growth factor beta helps regulate neuronal survival, neurogenesis, synaptogenesis, and gliogenesis (Figure 1) (88, 89). Astroglia expression of TGF-β mediates synaptic refinement as well as glial scar formation (90, 91). Abnormal TGF-β hyperactivation has been detected in neurodegenerative disease and traumatic injury patients, and astrocytes and microglia are the predominate source (92–95). Studies *in vitro* have shown that TGF-β may promote cell survival since supplementing TGF-β protects neurons from β-amyloid toxicity (96). This protective activity was further demonstrated to be antagonized by β-amyloid (97). Furthermore, the expression of the TFG-β type II receptor, mainly expressed by neurons, is reduced in AD brains (72). Therefore, it is clear that TGF-β has both beneficial and detrimental effects. Further work is necessary to determine when TGF-β becomes detrimental in response to neurodegenerative disease.

## Astrocyte Biomarkers in Alzheimer's Disease

Reactive astrocytes have become a focus of study in neurodegenerative disease and are essential players in the pathological process of AD and suggested to be targeted for novel therapeutics (34). Typically, immunohistochemical markers for reactive astrocytes are cytoskeletal components such as GFAP, vimentin, and nestin (98). However, the elevated marker such as GFAP alone is insufficient in categorizing astrocytes as reactive (37). Therefore, multiple markers are necessary to classify astrocytes as reactive.

Alzheimer's disease is classically diagnosed based on clinical criteria while the gold standard of definitive diagnosis is via neuropathology. Diagnosis based on clinical symptoms has a 30% misdiagnosis rate in comparison to neuropathological diagnosis (99). Thus, significant effort has been dedicated to develop clinical tools and tests to establish accurate early diagnosis and monitor the progression of the disease. Initial investigation for CSF markers began with classic astrocyte biomarkers such as GFAP, S100B, and glutamine synthetase, which proved to be not specific candidates due to their associations with homeostatic states of astrocytes and multiple other neurodegenerative diseases (100). Growing research has focused on a new astrocyte CSF biomarker Chitinase-3 protein like-1 (YKL-40), a protein commonly measured as a surrogate marker of neuroinflammation in AD (Figure 1) (101, 102). It has been linked to predict progression from normal cognition to mild cognitive impairment (MCI) and MCI to AD (102). Furthermore, elevated CSF YKL-40 has been confirmed to be correlated with phosphorylated tau levels at the early stages of AD (103). However, YKL-40 is not specific to AD alone and can be elevated in other tauopathies. Thus, further evaluation with multiple reactive astrocyte biomarkers is likely required to test for accuracy and progression of the disease.

Imaging modalities such as Positron emission tomography (PET) imaging and Magnetic resonance imaging (MRI) can assess aberrant astrocyte metabolism and detect accelerated brain atrophy. Currently, an inhibitor of the enzyme monoamine oxidase B (MAOB) [11C]-deuterium-*L*-deprenyl has been proposed as a PET imaging biomarker of reactive astrocytes (62, 104–106). MAOB is known to be up-regulated in GFAP-immunoreactive astrocytes. In post-mortem samples of individuals afflicted with AD, both the activity of MAOB and binding of *L*-deprenyl was found to be increased in multiple areas of the cerebral cortex (107). Furthermore, Gulyas et al. demonstrated that the highest binding of *L*-deprenyl occurs in the initial stages of AD (108). This suggests *L*-deprenyl as a promising PET imaging biomarker in the early diagnosis of AD.

The Alzheimer's precision medicine initiative was formed to review current blood-based AD biomarkers (99, 109). At this time no significant blood-based biomarker has proven to be effective. Therefore, it is crucial to continue investigating and elucidate additional biomarkers for treatment/diagnosis and signaling pathways to target for a better understanding of disease progression. It is unlikely a single biomarker will be found for diagnostic purposes, but rather reactive astrocyte signatures will be required to increase the specificity of diagnostic tests (37).

## Heterogeneity of Reactive Astrocytes in Alzheimer's Disease

Most studies of AD focus on tissue samples that contain many cell types. However, this cannot distinguish the contribution of specific cell types in AD. Although astrocytes could be isolated from Alzheimer's samples based on specific cell markers (110), the functions of each subpopulation are not clear due to the heterogeneity of astrocytes. Most of the current studies for astrocytes emphasize two morphological groups: fibrous and protoplasmic astrocytes, located in the white and gray matter of the brain, respectively (111, 112). However, how many astrocyte subclusters/subpopulations in different regions of the brain and what functions they play are not clear. Recently, the single-cell (scRNA-seq) or single-nucleus RNA-seq (snRNA-seq) methods have been developed and make it possible to analyze cell subtypes or status (113). For human Alzheimer studies, most of the time, only post-mortem frozen samples are available. Single-nucleus RNA-seq is an effective method to analyze individual cells using these samples. Grubman et al. obtained 13,214 high-quality nuclei of entorhinal cortex samples from control and Alzheimer's disease brains (114). Astrocytes (2,171 nuclei) were clustered into eight groups (a1–a8) by bioinformatic analysis. The functional enrichment showed astrocyte subpopulations might have different functions. For example, a1 astrocyte subpopulation was enriched in genes linked to ribosomal and mitochondrial function neuron differentiation and heat shock responses; a2 was enriched in transforming growth factor TGF-β signaling and immune response; a3 and a8 were enriched in cellular responses to lipids and hormones; a4 was enriched in respiratory and mitochondrial genes, whereas a6 was enriched in synapse organization, action potentials, and ion channel activity. Mathys et al. isolated single-nucleus from 48 post-mortem human prefrontal cortex samples (24 individuals with high levels of β-amyloid and other pathological hallmarks of AD, and 24 individuals with no or very low β-amyloid burden or other pathologies) (115). A total of 80,660 droplet-based single-nuclei was sequenced and was used for identifying transcriptionally distinct subpopulations. Three thousand three hundred and ninety-two astrocytes (1,562 cells from no-pathology individuals and 1,830 cells from AD-pathology individuals) were clustered into 4 AD-associated subpopulations (Ast0–Ast3), which were related to the different pathological features of source brains. For example, Ast1 was associated with a high amyloid level, high Braak stage (V), low CERAD (Consortium to Establish a Registry for AD) score, low NIA (National Institute on Aging)-Reagan score, and pronounced cognitive decline, while Ast0 was associated with no pathological traits. They also found astrocyte subpopulations have different responses to AD pathology between female and male individuals: Ast1 was enriched in female cells, whereas Ast0 was enriched in male cells. Zhou et al. analyzed 66,311 individual nuclei from dorsolateral prefrontal cortexes, and found six sub-clusters (Astro0–5) in control (2,955 astrocytes) and AD (2,641 astrocytes) samples (116). Compared with control, Astro3 was depleted in AD. Genes related to the coordination of lipid and oxidative metabolism between neurons and astrocytes, such as *FABP5, HILPDA*, and *SOD2*, were down-regulated in AD samples; while the expression of *NCAN* and *COL5A3*, which had functions on the extracellular matrix, were up-regulated in Astro0 and Astro1. These results suggested AD astrocytes might have lost metabolic coordination with neurons in AD. Additionally, Lau et al. sequenced 169,496 nuclei from prefrontal cortical samples of 12 AD patients and nine normal control (NC) subjects (117). From these samples, 17,997 nuclei were of astrocyte origin. The subcluster analysis showed that astrocytes were grouped into nine clusters (a1–a9). The proportion of cells in each subpopulation revealed the relative proportion of a2, a4, a5, a7, a8, and a9 were similar between AD and NC samples. However, the proportion of a1 and a6 were 9.9 and 10.2% larger and a3 were 23.5% smaller in AD, compared to the NC samples. The differential expressed genes across conditions in a1, a3, and a6 demonstrate the DEGs in a1 and a6 were enriched in up-regulated genes and a3 were enriched in down-regulated genes in AD samples. The enriched genes in a1 and a6 were associated with stress response genes, while genes in a3 were associated with neurotransmitter metabolism. All the above results suggest astrocytes from different brain regions might have specific astrocyte subpopulations. These subpopulations can be related to different AD pathology.

Regarding animal models of AD, 5xFAD transgenic mice are commonly used (118). Habib et al. analyzed 54,769 single-nucleus RNA-seq profiles from eight 7-month male mice hippocampus [four WT mice and four transgenic models of AD (5xFAD) mice] to define the role of non-neuronal cells in AD progression (119). Seven thousand three hundred and forty-five WT and AD astrocytes were clustered into six subclusters. A continuous trajectory across astrocyte subclusters showed three end states [*Gfap*-low, *Gfap*-high, and DAA (disease-associated astrocyte, a specific cluster in AD compared with WT)]. In these six clusters, clusters 1 and 2 were *Gfap*-low states and cluster 6 was *Gfap*-high state astrocytes; cluster 5 might be the transitional-like intermediate state between the *Gfap*-low state and *Gfap*-high state; cluster 3 might be a transitional-like intermediate state between *Gfap*-low stage and the DAA (cluster 4). Exemplary studies of astrocyte heterogeneity outlined above is outlined in Table 1. The continuous expression spectrum suggested astrocytes have a dynamic activation process in AD.

**Table 1:** Example studies of astrocyte heterogenity in AD.

**Species**	**Reference**	**Tissue**	**Total nuclei no**.	**Astrocyte nuclei no**.	**Astrocyte clusters**
Human	Grubman et al. (114)	Entorhinal cortex	13,214	2,171	a1–a8
Human	Mathys et al. (115)	Prefrontal cortex	80,660	3,392	Ast0-Ast3
Human	Zhou et al. (116)	Prefrontal cortex	66,311	Control (2,955) and AD (2,641)	Astro 0-5
Human	Lau et al. (117)	Prefrontal cortex	169,496	17,997	a1–a9
Mouse (5xFAD)	Habib et al. (119)	Hippocampus	54,769	7,345	Cluster 1–6

As astrocyte isolation is challenging, the proportion of astrocytes obtained in total cells is around 10% in the published papers. Also, the current single-cell technologies are limited with low number of transcripts per nuclei/cell compared to bulk RNA-seq. Future improvement of technology and astrocyte isolation will enhance our understanding of astrocyte heterogeneity in AD.

## Discussion

In summary, there is overwhelming evidence of the vital role astrocytes play in the pathophysiological development and progression of AD. The advent of technologies such as single-cell RNA sequencing and single-molecule imaging provides a greater understanding of the temporal and spatial progression of astrocytes that occurs during AD, which could serve as a framework for researchers to elucidate specific astrocytic biomarkers involved in AD progression (120). Specifically, studies using transcriptomics have allowed us to understand further that reactive astrocytes develop different molecular states during the progression of AD (37). As mentioned earlier, scRNAseq in AD models has demonstrated multiple stage-dependent conditions or subpopulations of reactive astrocytes (114–116). These studies signify the importance of characterizing the complex diversity and function of reactive astrocytes in each individual state to understand further the unique role these changes have in AD progression (114, 115). Therefore, it is not as simple to classify reactive astrocytes in AD as protective or toxic. Understanding the molecular changes at a single-cell level could also provide insight on the time point in which therapeutic intervention against reactive astrocytes can be applied, to harness AD progression and symptoms. The combination of powerful technologies such as viral gene transfer, electrophysiology, and optogenetics with transcriptomics can further elucidate the functions of reactive astrocytes in AD (37, 121).

Additionally, the roles and mechanisms of regulatory RNAs, such as long non-coding RNAs (lncRNAs), are underexplored in AD (122, 123). Currently, studies have demonstrated the regulatory role of lncRNA as it relates to tau hyperphosphorylation and others have suggested the utility of lncRNA as a biomarker for AD (124). These studies have provided the exciting potential of lncRNA as both diagnostic and therapeutic targets for AD.

Another critical consideration in elucidating the pathophysiological mechanisms of AD and determining fruitful therapeutic targets is ensuring we select appropriate *in vitro* and *in vivo* study models. Most cellular spatial information regarding cellular relationships to β-amyloid and neurofibrillary tangles is lost when isolating mRNA samples (125). Similarly, morphological and transcriptomic comparisons on human and mouse reactive astrocytes have revealed significant differences (37). This exemplifies the inherent limitations of *in vitro* studies and animal models in AD, and the difficulty in interpreting results when comparing studies with post-mortem specimens. Human induced pluripotent stem cells are currently increasingly employed in basic science research and can help narrow these differences (37). Furthermore, using multiple genomic techniques in combination, such as spatial transcriptomics and *in situ* sequencing, provides a benefit in preserving cellular spatial information (125). In conclusion, a consensus regarding appropriate research models and the integration of multiple “omic” modalities could provide improved diagnostic and therapeutic targets in reactive astrocytes.

## Author Contributions

All authors listed have made a substantial, direct and intellectual contribution to the work, and approved it for publication.

## Conflict of Interest

The authors declare that the research was conducted in the absence of any commercial or financial relationships that could be construed as a potential conflict of interest.

## Publisher's Note

All claims expressed in this article are solely those of the authors and do not necessarily represent those of their affiliated organizations, or those of the publisher, the editors and the reviewers. Any product that may be evaluated in this article, or claim that may be made by its manufacturer, is not guaranteed or endorsed by the publisher.
